# The effect of TLR3 priming conditions on MSC immunosuppressive properties

**DOI:** 10.1186/s13287-023-03579-y

**Published:** 2023-11-29

**Authors:** Tatiana Tolstova, Ekaterina Dotsenko, Peter Kozhin, Svetlana Novikova, Victor Zgoda, Alexander Rusanov, Nataliya Luzgina

**Affiliations:** Institute of Biomedical Chemistry, Pogodinskaya, Moscow, Russia 119121

**Keywords:** MSCs, Toll-like receptors, TLR3, Poly(I:C), Immunosuppression, Jurkat

## Abstract

**Background:**

Mesenchymal stromal cells (MSCs) have regenerative and immunomodulatory properties, making them suitable for cell therapy. Toll-like receptors (TLRs) in MSCs respond to viral load by secreting immunosuppressive or proinflammatory molecules. The expression of anti-inflammatory molecules in MSCs can be altered by the concentration and duration of exposure to the TLR3 ligand polyinosinic-polycytidylic acid (poly(I:C)). This study aimed to optimize the preconditioning of MSCs with poly(I:C) to increase immunosuppressive effects and to identify MSCs with activated TLR3 (prMSCs).

**Methods:**

Flow cytometry and histochemical staining were used to analyze MSCs for immunophenotype and differentiation potential. MSCs were exposed to poly(I:C) at 1 and 10 μg/mL for 1, 3, and 24 h, followed by determination of the expression of *IDO1*, *WARS1*, *PD-L1*, *TSG-6*, and *PTGES2* and PGE2 secretion. MSCs and prMSCs were cocultured with intact (J^−^) and activated (J^+^) Jurkat T cells. The proportion of proliferating and apoptotic J^+^ and J^−^ cells, IL-10 secretion, and IL-2 production after cocultivation with MSCs and prMSCs were measured. Liquid chromatography–mass spectrometry and bioinformatics analysis identified proteins linked to TLR3 activation in MSCs.

**Results:**

Poly(I:C) at 10 μg/mL during a 3-h incubation caused the highest expression of immunosuppression markers in MSCs. Activation of prMSCs caused a 18% decrease in proliferation and a one-third increase in apoptotic J^+^ cells compared to intact MSCs. Cocultures of prMSCs and Jurkat cells had increased IL-10 and decreased IL-2 in the conditioned medium. A proteomic study of MSCs and prMSCs identified 53 proteins with altered expression. Filtering the dataset with Gene Ontology and Reactome Pathway revealed that poly(I:C)-induced proteins activate the antiviral response. Protein‒protein interactions by String in prMSCs revealed that the antiviral response and IFN I signaling circuits were more active than in native MSCs. prMSCs expressed more cell adhesion proteins (ICAM-I and Galectin-3), PARP14, PSMB8, USP18, and GBP4, which may explain their anti-inflammatory effects on Jurkat cells.

**Conclusions:**

TLR3 activation in MSCs is dependent on exposure time and poly(I:C) concentration. The maximum expression of immunosuppressive molecules was observed with 10 µg/mL poly(I:C) for 3-h preconditioning. This priming protocol for MSCs enhances the immunosuppressive effects of prMSCs on T cells.

**Supplementary Information:**

The online version contains supplementary material available at 10.1186/s13287-023-03579-y.

## Introduction

The unique properties of MSCs determine their potential use in cell therapy as well as the relevance of developing biotechnological preparations based on the secretome of these cells. MSCs are actively involved in tissue repair after damage by regulating cell proliferation, migration, and differentiation owing to their high paracrine activity [1, 2].

The ability of MSCs to induce immunosuppression has been previously demonstrated. MSCs reduce the number of proliferating T cells [3] by stopping the G0/G1 phase of the cell cycle [4], regulating the T-helper type 1/2 (Th1/Th2) balance [5], promoting the formation of B-regulatory cells [6], and modulating the behavior of natural killer (NK) cells, dendritic cells, etc. [7]. MSCs are approved for the treatment of Crohn's disease (Daradstrocel, Alofisel) [8], acute graft-versus-host disease (TEMCELL) [8], etc.

Experimental approaches are currently being developed to enhance the immunosuppressive properties of MSCs. Thus, in several studies, preincubation with IFN-γ was used for this purpose [9–12]. An increase in the immunosuppressive properties of MSCs is also noted under conditions of “physiological” hypoxia: the secretion of anti-inflammatory cytokines of MSCs (IL-10, TGF-β) as well as the ability to suppress activation and T-cell proliferation [13, 14].

Recently, a new strategy for modulating the immunoregulatory properties of MSCs by priming Toll-like receptors has been actively discussed [15]. It is assumed that this approach will make it possible to obtain MSC-based cell products more homogeneous and to achieve higher clinical efficiency by providing cells with a target functional status. In particular, according to some authors, TLR3 activation enhances the immunosuppressive activity of MSCs [15–18]. At the same time, MSCs do not acquire immunogenicity: Lombardo *et al*. showed that, unlike IFN-γ, priming of cells with TLR ligands does not lead to an increase in HLA-II expression [16].

However, the modes of TLR3 MSC priming used by different authors vary significantly with the applied concentrations and time of exposure to TLR ligands. As a result, the phenotype of MSCs with activated TLRs, including their ability to immunosuppress, differs. Thus, short-term priming (up to 24 h) with low (up to 10 µg/mL) [17] or high (up to 50 µg/mL) [19] doses of TLR3 ligand resulted in increased secretion of anti-inflammatory factors and more pronounced immunosuppressive effects on T cells. However, prolonged activation (up to 72 h) with a low (1 μg/mL) concentration of poly(I:C) had no effect on the immunosuppressive properties of MSCs. Moreover, priming for 48 h with a high concentration of ligand (20 μg/mL) enhanced the proinflammatory properties [20].

In general, the functional features of MSC subpopulations with activated TLRs, and consequently, the specifics of their therapeutic potential, are currently not fully understood.

The aim of this study was to select the optimal protocol for TLR3 priming of human adipose-derived MSCs to enhance their immunosuppressive properties and phenotypic characterization of the subpopulation of MSCs with activated TLR3.

## Methods

### MSCs expansion

The total pool (three individual donors) of human adipose-derived MSCs was purchased from the cryobank of the Perspectiva Research-and-Production Company (Novosibirsk). MSCs were cultured in DMEM/F12 supplemented with 10% FBS, GlutaMAX^TM^ and 1% penicillin/streptomycin (all from Gibco) until 70–80% confluence was reached. After that, the cells were treated with 0.25% trypsin (Gibco) and reseeded at 5–7 × 10^3^ cells/cm^2^ in a T175 tissue culture flask (Corning). MSCs that were not older than passage five were selected for the study.

### MSCs differentiation

MSCs were cultured in a 6-well plate (Corning) in induction medium, as described early [21]. Briefly, for MSCs differentiation into osteoblasts, DMEM/F12 culture medium with the addition of osteogenic supplements (100 nM dexamethasone, 10 mM β-glycerol phosphate, and 0.05 mM ascorbate-2-phosphate) was used. The addition of 1 μg/mL insulin, 0.5 mM 3-isobutyl-1-methylxanthine, and 0.5 μM dexamethasone to the growth medium stimulated adipogenic differentiation (all from Sigma‒Aldrich). In the control wells, MSCs were cultivated in growth medium without inducers. The medium was refreshed every 96 h. Alizarin red and oil red O (all from Sigma‒Aldrich) staining was used to assess osteogenesis and adipogenesis, respectively, on the 14th day of cultivation by phase-contrast microscopic observation (a Primovert microscope, Carl Zeiss).

### TLR3 priming protocol

To obtain prMSCs, cells were seeded at a density of 5–7 × 10^3^ cells/cm^2^. After 24 h, the medium was replaced with fresh medium containing a TLR3 agonist. We used poly(I:C) (Sigma‒Aldrich, Cat# P1530) at concentrations of 1 or 10 μg/mL for the induction of prMSCs. The cells were cultured with the inductor for 1, 3, or 24 h to determine the most efficient activation protocol. The control was cultivated on the growth medium at the same time intervals. The cells were then washed three times with PBS (Gibco) and incubated in complete culture medium for 2 or 24 h to obtain cell lysates (for quantitative real-time PCR analysis (qRT‒PCR)) or for 24 h to obtain conditioned medium (for enzyme-linked immunosorbent assay (ELISA)) and proteomic analysis.

### Flow cytometry analysis

MSCs were verified for CD105- (Cloud-Clone Corp., Cat# FAA980Hu82), CD90- (Abcam, Cat# ab23894), and CD73-positive antibody staining (Cloud-Clone Corp., Cat# LAB250Hu81), and negative staining for CD45 (Abcam, Cat# ab200315), CD34 (Abcam, Cat# ab157325) and HLA-DR (Thermo Fisher Scientific, Cat# 45995671). TLR3 expression was confirmed using anti-TLR3 monoclonal antibody staining (Abcam, Cat# ab45093). PD-L1 expression was confirmed using anti-PD-L1 polyclonal antibody staining (Cloud-Clone Corp., Cat# LAB250Hu81).

MSCs (5 × 10^5^ cells) were suspended in PBS and incubated with the monoclonal antibodies described above and isotype control antibodies (Bio-Rad, Cat# MCA928). After incubation, the excess antibodies were washed with PBS at 300 × g for 5 min. The cells were then resuspended in PBS (0.5 mL) and analyzed using a ZE5 Cell Analyser (Bio-Rad).

### MSCs/prMSCs immunosuppression assay

#### qRT‒PCR

RNA from MSCs and prMSCs was isolated using the RNeasy Mini Kit (Qiagen). The quantity and purity were determined using a NanoDrop spectrophotometer (Thermo Fisher Scientific). Reverse transcription was carried out using an MMLV RT kit (Evrogen). Real-time qRT‒PCR was performed using the qPCRmix-HS SYBR+LowROX kit (Evrogen) in the CFX96 real-time PCR Detection System instrument (Bio-Rad Laboratories). Primer sequences are provided in Additional file 1: Table S1. Data were analyzed using the efficient ∆∆Ct method, with *ACTB* and *GAPDH* as reference genes. All samples were run in triplicate. Data represent the mean ± SEM from three independent experiments. Raw data were analyzed using CFX Maestro 1.0 software (v. 4.0.0325.0418).

#### ELISA

Cytokines in conditioned media were assayed using Interleukin-2-EIA-BEST ELISA kits (Vector-Best, Cat# 8772), Interleukin-10-EIA-BEST ELISA kits (Vector-Best, Cat# 8774) and Prostaglandin E2 Express ELISA Kits (Cayman Chemical Co., Cat# 500141) according to the manufacturer’s recommendations. Data represent the mean ± SEM from three independent experiments performed in triplicate.

#### Coculture protocol

Jurkat cells (Institute of Cytology RAS, Saint-Petersburg) were cultured in RPMI-1640 medium supplemented with 10% FBS, GlutaMAX^TM^ and 1% penicillin/streptomycin (all from Gibco) at 37 °C with 5% CO_2_. Jurkat cells were activated with 10 ng/mL phorbol myristyl acetate (PMA) and 1 μg/mL phytohemagglutinin (PHA) (all from Sigma) for 24 h. MSCs were cultured in a 6-well plate (7 × 10^4^ cells/well), primed with 10 μg/mL poly(I:C) for 3 h and washed three times with PBS. Control wells were similarly treated without exposure to the TLR3 agonist. The cells were then incubated in complete culture medium for 24 h. The nonactivated and activated Jurkat cells were washed with PBS and added to MSCs and prMSCs at a ratio of 10:1, respectively. After 24 h of coculture, the conditioned medium was collected for ELISA, and Jurkat cells were used for qRT‒PCR and flow cytometric analysis.

#### Cell cycle analysis

Cocultured and intact Jurkat cells were incubated with EdU (Invitrogen^TM^) for 1 h before harvesting. Non-cocultured Jurkat cells (nonactivated or activated) were used as the control. The cells were washed with PBS and fixed with 4% (v/v) neutral-buffered formalin (Thermo Fisher Scientific) for 1 h. The Click-iT® Plus EdU Alexa Fluor® 488 Flow Cytometry Assay Kit (Invitrogen^TM^, Cat# C10633), following the manufacturer's instructions, was used for cell cycle analysis. The cell cycle distribution was analyzed using flow cytometry (ZE5 Cell Analyser, Bio-Rad). Floreada.io software was used for data analysis (https://floreada.io/analysis).

#### Apoptosis assay

The effect of coculturing Jurkat cells with MSCs or prMSCs on the apoptotic cell count was assessed using Annexin V-FITC and propidium iodide (PI) (Invitrogen^TM^, Cat# V13242). Briefly, Jurkat cells were harvested from MSCs/prMSCs, washed with PBS, and resuspended in annexin binding buffer. Jurkat cells were stained with Annexin V and PI for 15 min in the dark. Next, the cells were washed twice with annexin binding buffer and analyzed by flow cytometry (ZE5 Cell Analyser, Bio-Rad).

### Proteome analysis

#### Protein extraction and in-solution digestion

After treatment with trypsin, the MSCs and prMSCs were washed three times with PBS. Approximately 0.7 x 10^6^ cells were used for protein extraction. The cells were lysed in 300 μL of buffer containing 3% sodium deoxycholate and 0.1 M Tris-HCl (pH 7.6) on ice for 30 min. To reduce the viscosity of the solution, short-term sonication was performed using a Bandelin Sonopuls probe («BANDELIN Electronic GmbH&Co. KG», Germany, Berlin). Total protein was quantified by the colorimetric method using a Pierce™ BCA Protein Assay Kit (Pierce, Rockford, IL, USA) in accordance with the manufacturer’s recommendations. Each sample (50 µg) was subjected to in-solution digestion, as described previously [22], with slight modifications.

Briefly, one-step disulfide bond cleavage, combining reduction and alkylation, was performed in the presence of 50 mM Tris(2-carboxyethyl) phosphine (Thermo Fisher Scientific) and 80 mM chloroacetamide (Sigma‒Aldrich) in 50 mM triethylammonium bicarbonate buffer (TEAB) (Sigma‒Aldrich) (pH 8.5) at 80 °C within 1 hour. To dissolve the reaction mixture, 100 µL of 50 mM TEAB was added to each sample. Sequencing grade trypsin (Sequencing Grade Modified, Promega, Madison, WI, USA) was added to each sample at an “enzyme:protein” ratio of 1:50, followed by overnight incubation at 37 °C. Formic acid (Sigma‒Aldrich) was added to the quenched hydrolysis to a final concentration of 5%. To clarify the peptide solution, the samples were centrifuged at 14,000×*g* for 15 min. The resulting supernatant was used for subsequent MS analysis. Prior to LC‒MS/MS, peptide concentrations were measured by the colorimetric method using the Pierce™ Peptide Quantitative Colorimetric Assay Kit (Pierce, Rockford, IL, USA) according to the manufacturer's recommendations. The peptides were dried and dissolved in 0.1% formic acid to a final concentration of 2 μg/μL.

#### Liquid chromatography–mass spectrometry analyses (LC‒MS/MS)

Each sample containing 1 μg of total peptides was loaded onto an Acclaim μ-precolumn (0.5 mm × 3 mm, particle size 5 μm, inner diameter 75 μm; Thermo Scientific) and washed with mobile phase C (2% acetonitrile, 0.1% formic acid in HPLC-grade water) at a flow rate of 10 μL/min for 4 min. The peptides were separated on an analytical column Acclaim® PepMap^TM^ RSLC 75 µm ID (Thermo Fisher Scientific) using a gradient of mobile phase A (0.1% formic acid in HPLC-grade water) and mobile phase B (80% acetonitrile, 0.1% formic acid in HPLC-grade water). The total run time was 130 min, which included 12 min of column equilibration with mobile phase A, a gradient from 5 to 35% mobile phase B over 95 min, 6 min to reach 99% mobile phase B, 10 min of washing with 99% mobile phase B and re-equilibration with mobile phase A for 7 min.

MS/MS analysis was performed using a Q Exactive HF-X mass spectrometer (Q Exactive HF-X Hybrid Quadrupole-Orbitrap^TM^ Mass spectrometer, Thermo Fisher Scientific). The ion source was operated at a capillary temperature of 240 °C and an emitter voltage of 2.1 kV. The MS mass spectrum acquisition was performed at a resolution of 120,000 at *m/z* = 400 in the mass range of 300–1500 *m/z*. Tandem mass spectra of the fragments were acquired at a resolution of 15,000 at *m/z* = 400 in the mass range of 140–2000 *m/z*. The AGC target was set at 1 × 10^6^ and 2 × 10^5^, with maximum ion injection times of 50 ms and 110 ms for precursor and fragment ions, respectively. Up to 20 precursors with an intensity threshold of 50,000 counts were chosen to trigger the MS/MS spectra. High-energy collisional dissociation was applied with a normalized collision energy set at 29 V. Precursors with a charge state of +1 and more than +5 were omitted, and all precursors that were already fragmented in the current work cycle were dynamically excluded from triggering a subsequent MS/MS for 20 s.

#### Data analysis

For identification and label-free quantification, mass spectrometry data were loaded into MaxQuant software (version 1.6.0.16, Max Planck Institute of Biochemistry, Martinsried). The proteins were identified using the built-in Andromeda algorithm. Identification was carried out using the FASTA file (UniProt release 15-04-2022, EMBL-EBI, Hinxton Cambridge) and its inverted counterpart to calculate the frequency of false positive identifications (FDR), along with a built-in database of potential contaminants. Carbamidomethylation of cysteine was used as a fixed modification, and methionine oxidation and N-terminal acetylation were used as variable modifications. The tolerance of the precursor and fragment ions was 20 ppm. The FDR threshold value for proteins and peptides was 0.01. Quantitative analysis was carried out based on the area under the peak of the parent ion with calculation of the label-free quantification (LFQ) value performed using the algorithm built into MaxQuant (version 1.6.0.16, Max Planck Institute of Biochemistry, Martinsried) [23]. Unique peptides without modifications were used for quantitative assessment. Potential contaminants, false positive identifications, and proteins identified only by peptides containing modifications were removed from the potentially identified proteins.

The statistical analysis was performed using Perseus 1.6.0.7 software (Max Planck Institute of Biochemistry, Martinsried, Germany). A two-sample t test was used to compare the two groups. The FDR threshold value for permutation (correction for multiple comparisons) was 0.05, S0 = 0.1. We compared the proteins for which at least two unique peptides per protein were identified.

The STRING database v.11.0 was used to retrieve the protein–protein interactions from the lists of MSC proteins with differential expression. A high confidence score (0.7) was applied. Active interaction sources were text mining, experiments, and databases. Functional enrichment analysis was performed using Panther and Gorilla online tools. The annotation sources were the “Biological process” category of the Gene Ontology (GO) database and the Reactome pathway database. The full set of canonical forms of human proteins (20589 proteins according to Swiss-Prot) was used as the control sample. The capture cut-off (p value) was set at 10^−3^.

### Statistical analysis

Data are presented as the mean of triplicate samples and experiments. The differences between experimental groups were evaluated by one-way ANOVA with a Tukey’s post-test and by two-way ANOVA with a Sidak’s multiple comparisons test. Differences were considered statistically significant at *p* value < 0.05. All data are expressed as mean ± standard error of the mean (SEM). GraphPad Prism v8.0.1 was used for statistical analysis and graph design.

## Results

### Characterization of human adipose-derived MSCs

**Fig. 1:**
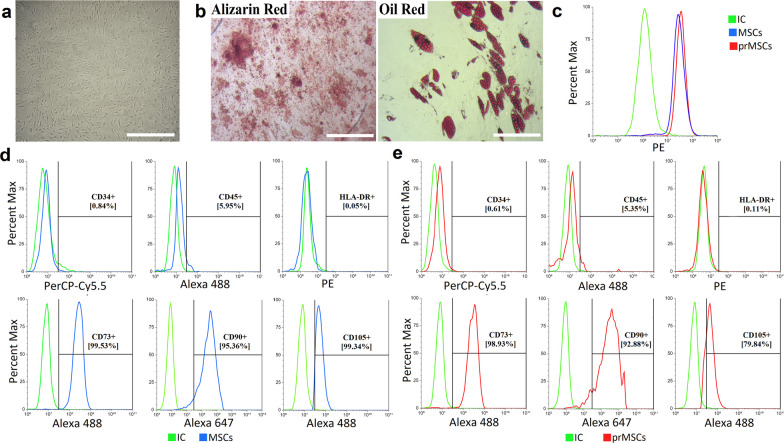
Phenotypic characterization of human adipose-derived MSCs. **a** The fibroblast-like morphology of adhesion cells was studied by phase-contrast microscopy. Scale 100 µm. **b** Multilineage differentiation of MSCs. Histochemical staining of calcium deposits (alizarin red) and neutral lipid accumulation (oil red O) after 14 days of osteogenesis and adipogenesis induction. Scale 100 µm. **c** Flow cytometry analysis of TLR3 expression: after 10 µg/mL poly(I:C) stimulation for 3 h – prMSCs (red line) and without stimulation – MSCs (constitutive expression, blue line), isotypic control—IC (green line). **d**, **e** Immunophenotypic characteristics of MSCs and prMSCs: negative expression of CD34, CD45, and HLA-DR markers (top panel); positive expression of CD73, CD90, and CD105 markers (bottom panel)

MSCs isolated from adipose tissue were characterized (Fig. 1) by plastic adherence (Fig. 1a) and multipotent differentiation (Fig. 1b), had a spindle-shaped morphology, were negative for the markers CD34, CD45, and HLA-DR, and were positive for CD73, CD105, and CD90 (Fig. 1d). This corresponds to the stem cell phenotype established by the International Society for Cell and Gene Therapy [24]. Priming of toll-like receptors, in particular TLR3, can confer immunomodulatory properties to MSCs [16, 17, 20, 25]. Flow cytometry analysis confirmed the high expression of TLR3 in MSCs as well as in prMSCs (Fig. 1c). Changes in concentration and exposure time did not affect TLR3 expression levels (data not shown). In addition, preincubation of MSCs with poly(I:C) did not lead to changes in the expression of surface markers, including HLA-DR (Fig. 1e).

### Evaluation of the influence of the TLR3 priming protocol on the production of immunosuppressive factors

**Fig. 2:**
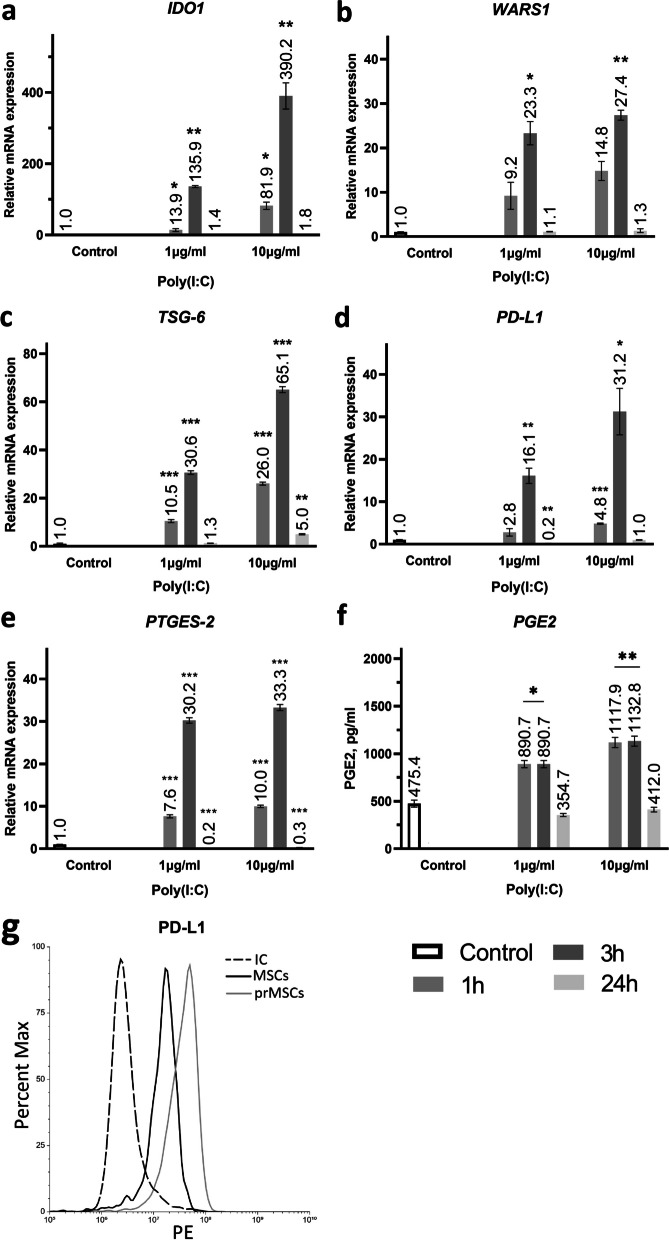
Evaluation of the influence of the TLR3 priming protocol on the production of immunosuppressive factors. Expression of the immunosuppressive effectors *IDO1* (**a**), *WARS1* (**b**), *TSG-6* (**c**), *PD-L1* (**d**) and *PTGES2* (**e**) genes, secretion of PGE-2 (**f**), activation of PD-L1 (**g**) in prMSCs cells. Control—unprimed MSCs. Data for various stimulation protocols: 1 μg/mL and 10 μg/mL poly(I:C) for 1,3 and 24 h (**a-e**). The analysis was performed 2 h (**c**–**e**) or 24 h (**a**, **b**, **f**, **g**) after preconditioning with poly(I:C). Error bars indicate SEM. *p < 0.05, **p < 0.01 and ***p < 0.005 compared to the control

The efficiency of TLR3 priming was determined by the expression of indoleamine 2,3-dioxygenase (*IDO1*)*,* tryptophanyl-tRNA synthetase 1 (*WARS1*), TNF-α–stimulated gene/protein 6 (*TSG-6),* Programmed cell death 1 ligand 1 (*PD-L1*), and prostaglandin-endoperoxide synthase 2 (*PTGES2*) genes by qRT‒PCR (Fig. 2a–e) and Prostaglandin E2 (PGE-2) secretion by ELISA (Fig. 2f). *IDO1* mediates the immunosuppressive effects of MSCs in an inflammatory environment or upon TLR3 priming [26, 27]. The increased expression of *WARS1* in MSCs may also affect their immunosuppressive properties [28]. It has been shown that preconditioning MSCs with poly(I:C) leads to an increase in the expression of the *IDO1* (Fig. 2a) and *WARS1* (Fig. 2b) genes after 24 h in a time- and dose-dependent manner: incubation of MSCs for 1 h with 1 μg/mL or 10 μg/mL poly(I:C) increased the *IDO1* expression level to 13.9 ± 4.0 or 81.9 ± 10.4, respectively, whereas *WARS1* expression increased to 9.2 ± 3.0 or 14.7 ± 2.1, respectively. Exposure to poly(I:C) for 3 h resulted in a significant increase in *IDO1* expression to 135.9 ± 3.0 and 390.2 ± 36.6 at 1 and 10 µg/mL, respectively. *WARS1* expression was also maximal after exposure to 10 µg/mL poly (I:C) for 3 h (27.4 ± 1.1-fold change). At the same time, long-term activation of TLR3 (24 h) in MSCs only slightly increased the expression of *IDO1* and *WARS1* up to 1.1 – 1.8 times.

Anti-inflammatory *TSG-6* is not known to be expressed in normal MSCs but is activated after exposure to TNF-α or other proinflammatory cytokines [29]. *TSG-6* expression in MSCs increased 2 h after preconditioning with poly(I:C) (Fig. 2c). The level of expression upon short-term stimulation of TLR3 was higher (fold change from 10.5 ± 0.6 to 65.1 ± 1.2) than that upon long-term (24 h) exposure to poly(I:C) (fold change up to 5.0 ± 0.2). A similar trend was observed for *PD-L1* expression (Fig. 2d). MSCs have been reported to express and secrete PD-L1 in a proinflammatory environment, which promotes MSC-mediated downregulation of CD4^+^ T-cell activation and decreased secretion of interleukin-2 by T cells [30]. It has been shown that the level of expression increases in a dose-dependent manner after short-term stimulation (fold change:2.8 ± 0.9 (1 h/1 µg/mL), 4.8 ± 0.1 (1 h/10 µg/mL), 16.1 ± 1.8 (3 h/1 µg/mL), 31.2 ± 5.5 (3 h/10 µg/mL)) and decreases with long-term exposure (fold change 0.2 ± 0.03 (24 h/1 µg/mL)) or does not change after 24 h preconditioning with 10 µg/mL poly(I:C). PD-L1 expression as a result of priming with 10 μg/ml poly(I:C) for 3 h was also confirmed by flow cytometry analysis (Fig. 2g).

PGE2 is constitutively secreted by MSCs, which is consistent with the results of ELISA (Fig. 2f) in our study, and plays a role in their inhibitory effect on dendritic cell differentiation and the proliferation of activated T cells [31]. The levels of *PTGES2* expression (Fig. 2e) after 2 h of preconditioning with TLR3 ligand were maximal as a result of 3 h of TLR3 activation with both 1 and 10 μg/mL poly(I:C). Fold changes in *PTGES2* expression were 30.2 ± 0.6 and 33.3 ± 0.7, respectively. The PGE2 concentrations (Fig. 2e) in the conditioned medium were 1117.9 ± 53.1 pg/mL and 1132.8 ± 52.6 pg/mL after incubation with 10 μg/mL poly(I:C) for 1 and 3 h, respectively.

Thus, the maximum production of anti-inflammatory factors was noted in MSCs after 3 h of preconditioning with 10 µg/mL poly(I:C). This protocol was chosen for subsequent studies on the immunosuppressive properties against Jurkat model T cells.

### TLR3 priming enhances MSC immunosuppression against T cells

**Fig. 3:**
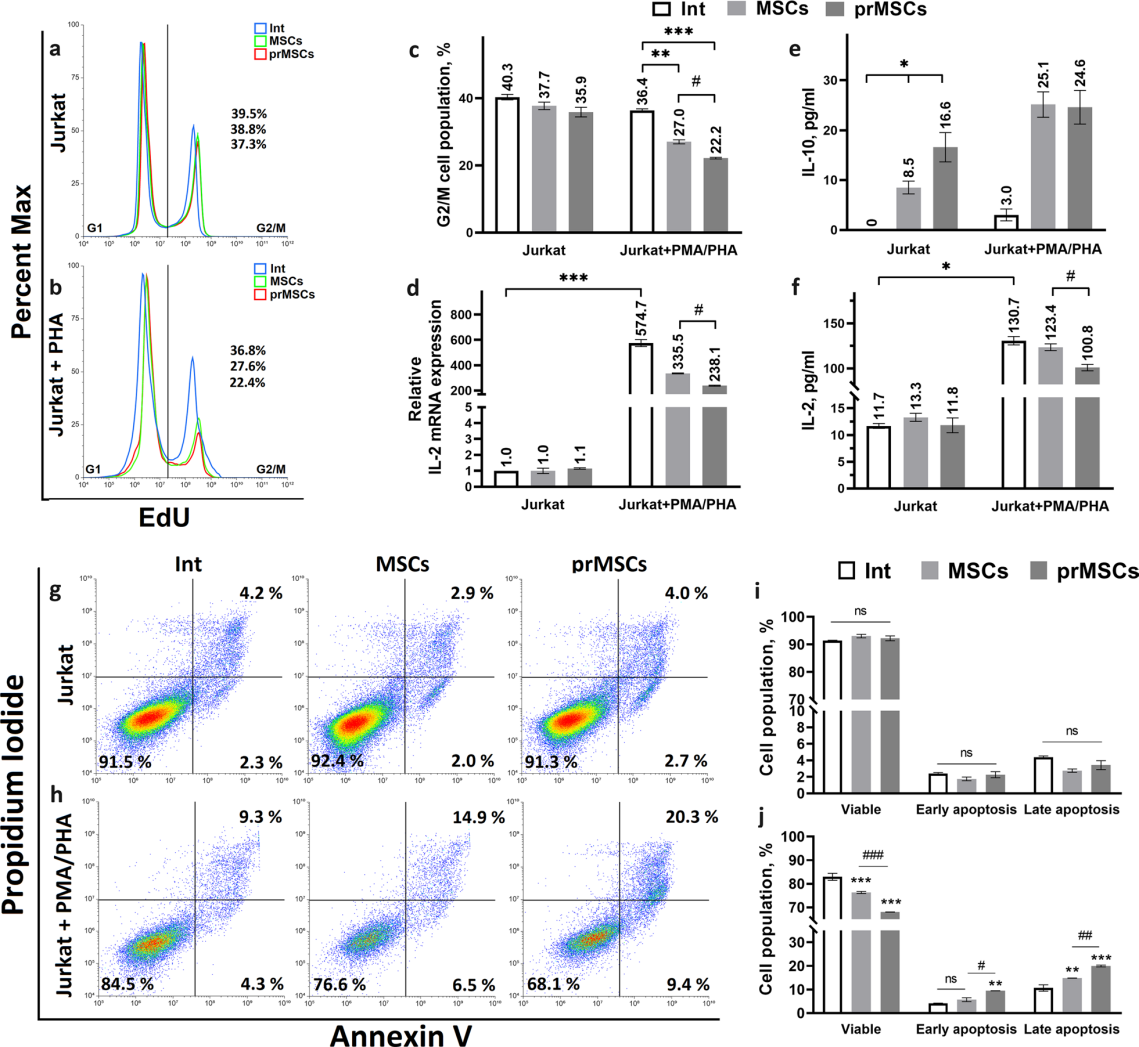
Influence of MSCs and prMSCs on activation (IL-2 production), the number of proliferating and apoptotic Jurkat cells, and the level of anti-inflammatory IL-10 cytokine. The number of cells in the G1-phase (left) and G2/M-phase (right) as a percentage of nonactivated Jurkat cells (**a**, **c**) and activated PMA/PHA Jurkat cells (**b**, **c**) (flow cytometry). Level of relative mRNA expression (**d**) and secretion (**f**) of IL-2 by Jurkat cells (qRT‒PCR and ELISA). Amount of cytokine IL-10 (**e**) in the conditioned medium (ELISA). The number of living cells (bottom right), cells in the early apoptosis stage (bottom left), and cells in the late apoptosis stage (top left) as a percentage of nonactivated Jurkat (**g**, **i**) and activated PMA/PHA Jurkat (**h**, **j**) (flow cytometry). Int—noncocultured cells, MSCs/prMSCs—after cocultivation with MSCs/prMSCs. Error bars indicate SEM. n.s.: not significant (*p* > 0.05); *p < 0.05, **p < 0.01 and ***p < 0.005 compared with the control. ^#^*p* < 0.05, ^##^*p* < 0.01 and ^###^*p* < 0.005 compared with the MSCs

The study of the immunosuppressive activity of MSCs and prMSCs was carried out on Jurkat cells, a human acute T lymphocyte leukemia cell line. Jurkat cells modulate T lymphocyte functions and are widely used in in vitro models [32]. The prMSCs were obtained by treatment with 10 μg/mL poly(I:C) for 3 h and incubated for 24 h in growth medium before coculture. Jurkat cells were nonactivated (J^−^) or preactivated for 24 h with 10 ng/mL PMA and 1 μg/mL PHA (J^+^). The J^−^ and J^+^ cells were added to the MSCs or prMSCs at a ratio of 10:1, respectively. After 24 h of coculture, the number of proliferating (Fig. 3a–c) and apoptotic (Fig. 3g–j) lymphocytes was examined by flow cytometry using a Click-iT® Plus EdU Assay Kit and Annexin V/propidium iodide, respectively. It has been shown that cocultivation with both MSCs and prMSCs leads to a decrease in the number of proliferating J^+^, but not J^−^ cells. The proportion of intact J^+^ cells in the G2/M-phase cell cycle was 36.4 ± 2.1%. After cocultivation of J^+^ with MSCs and prMSCs, the proliferating cell ratio was reduced to 27.0 ± 1.2% and 22.2 ± 0.8%, respectively. Thus, preconditioning MSCs with poly(I:C) made it possible to reduce the percentage of J^+^ cells in the G2/M-phase by 18% more efficiently than cocultivation of J^+^ cells with intact MSCs.

The data from the cell cycle analysis are in line with the results of Annexin V/PI staining (Fig. 3g–j). The rate of apoptotic cells did not change significantly between the groups in the J^−^ case (early apoptosis: 2.3%, 2.0% and 2.7%; late apoptosis: 4.2%, 2.9% and 4.0%, for intact or coculture with MSCs and prMSCs, respectively). At the same time, cocultivation of J^+^ with prMSCs led to an increase in the number of apoptotic cells up to 20.0 ± 0.6%, which is third more than that with MSCs (14.8 ± 0.4%).

In addition, MSCs can affect the activation of T cells by reducing the expression and secretion of IL-2. We have shown that Jurkat activation is associated with increased production of IL-2 (an increase in expression to 574.7 ± 4.1 and secretion to 130.7 ± 5.3 pg/mL). Cocultivation of J^+^ with MSCs and prMSCs reduced IL-2 expression to 335.5 ± 0.4 and 238.1 ± 0.7 and secretion to 123.4 ± 5.1 and 100.8 ± 4.3 pg/mL, respectively.

MSCs are known to increase the population of IL-10-producing T cells and affect IL-4-producing Th2 cells [33, 34]. IL-10 secretion was detected in intact J^+^ cells (Fig. 3e). The amount of IL-10 in the conditioned medium increased to approximately 25 μg/mL after the cocultivation of J^+^ with MSCs, regardless of the MSC phenotype. Although, in the case of J^−^, the total level of IL-10 was lower than that for J^+^, it differed between the coculture of J^−^ with MSCs and prMSCs by nearly 2 times (8.5 ± 1.3 pg/mL and 16.6 ± 3.0 pg/mL, respectively). The level of IL-4 (~ 4 pg/mL) in Jurkat cells did not change significantly after activation, preconditioning of MSCs and coculturing (data not shown).

Thus, cocultivation of Jurkat cells with prMSCs reduced the proliferative activity of J^+^ cells by 18%, the expression and secretion of IL-2 by 29% and 18%, respectively, and increased the proportion of apoptotic J^+^ cells by one-third in comparison to J^+^ cells cocultured with intact MSCs. In addition, the priming of MSCs affected the level of IL-10 when cocultured with nonactivated Jurkat cells. This confirmed the enhanced immunosuppressive properties of the prMSCs.

### LC‒MS/MS analyses of MSCs and prMSCs

#### Proteins Identified

As a result of mass spectrometric analysis of MSCs and prMSCs primed according to the protocol obtained (3 h, 10 µg/mL poly(I:C)), 1686 proteins were identified in all studied MSC samples by two or more peptides (potential contaminants, false positive identifications and proteins identifiable only by peptides containing modifications were excluded). Mass spectrometric data are available through ProteomeXchange with identifiers PXD044795.

**Fig. 4:**
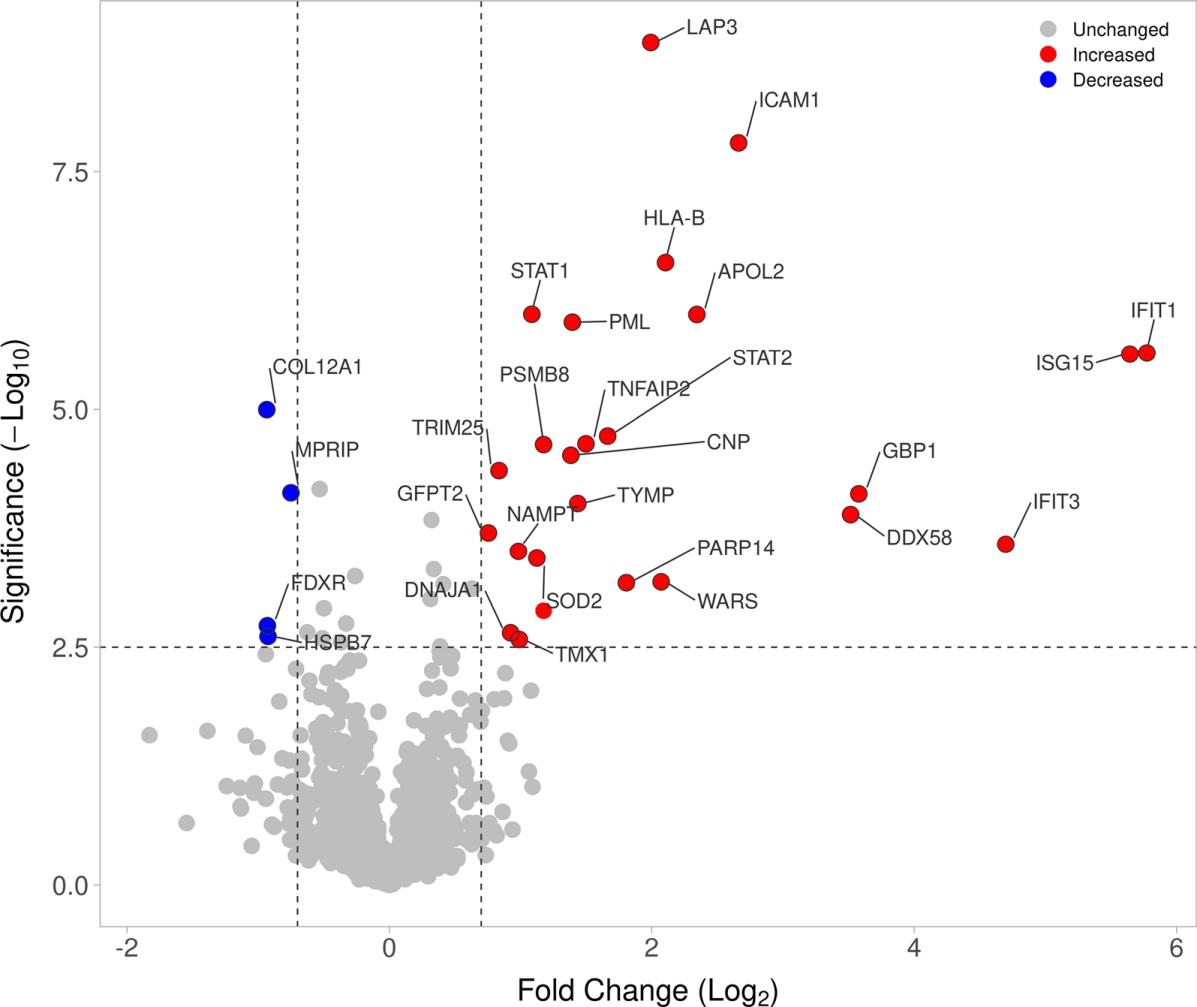
Volcano plot of the 1686 proteins identified by LC–MS/MS. Proteins with unchanged expression are marked in gray, those upregulated in prMSCs are marked in red, and those downregulated in prMSCs are marked in blue

A total of 53 proteins were identified as having changed expression. Among them, 24 and 4 proteins were upregulated and downregulated, respectively, in prMSCs, based on log2-fold change cut-off criteria, <  − 0.7 indicating downregulation and > 0.7 upregulation, with significance indicated by an adjusted *p* value < 0.05 (Fig. 4). A scatterplot (volcano plot) shows a measure of effect size versus a measure of significance for proteins that have changed expression in prMSCs. At the same time, 22 proteins were expressed de novo (Table 1), and 3 proteins were missed in prMSCs (Table 2).

**Table 1:** Proteins expressed de novo in prMSCs

Majority protein IDs	Protein names	Gene names
Q92692	Nectin-2	PVRL2
Q9NT62	Ubiquitin-like-conjugating enzyme ATG3	ATG3
P24539	ATP synthase F (0) complex subunit B1, mitochondrial	ATP5F1
Q9UMW8	Ubl carboxyl-terminal hydrolase 18	USP18
Q15646	2–5-oligoadenylate synthase-like protein	OASL
Q15056	Eukaryotic translation initiation factor 4H	EIF4H
P14902	Indoleamine 2,3-dioxygenase 1	IDO1
Q9Y6K5	2–5-oligoadenylate synthase 3	OAS3
O95793	Double-stranded RNA-binding protein Staufen homolog 1	STAU1
P36776	Lon protease homolog, mitochondrial	LONP1
Q10589	Bone marrow stromal antigen 2	BST2
Q08380	Galectin-3-binding protein	LG3BP
Q96PP9	Guanylate-binding protein 4	GBP4
Q96AZ6	Interferon-stimulated gene 20 kDa protein	ISG20
Q9H4M7	Pleckstrin homology domain-containing family A member 4	PLEKHA4
Q5EBM0	UMP-CMP kinase 2, mitochondrial	CMPK2
Q8TDB6	E3 ubiquitin-protein ligase DTX3L	DTX3L
P00973	2–5-oligoadenylate synthase 1	OAS1
Q9BYX4	Interferon-induced helicase C domain-containing protein 1	IFIH1
P20592	Interferon-induced GTP-binding protein Mx2	MX2
P20591	Interferon-induced GTP-binding protein Mx1	MX1
P09913	Interferon-induced protein with tetratricopeptide repeats 2	IFIT2

**Table 2:** Missing proteins in prMSCs

Majority protein IDs	Protein names	Gene names
Q9UBE0	SUMO-activating enzyme subunit 1	SAE1
P26196	Probable ATP-dependent RNA helicase DDX6	DDX6
Q71U36	Tubulin alpha-1A chain	TUBA1A

#### Overexpressed proteins and proteins identified in entirely prMSCs form an interaction cluster in STRING

The cluster of proteins lost in prMSCs and whose expression was reduced as a result of TLR3 activation consisted of 7 functionally isolated proteins (Fig. 4, Table 2).

**Fig. 5:**
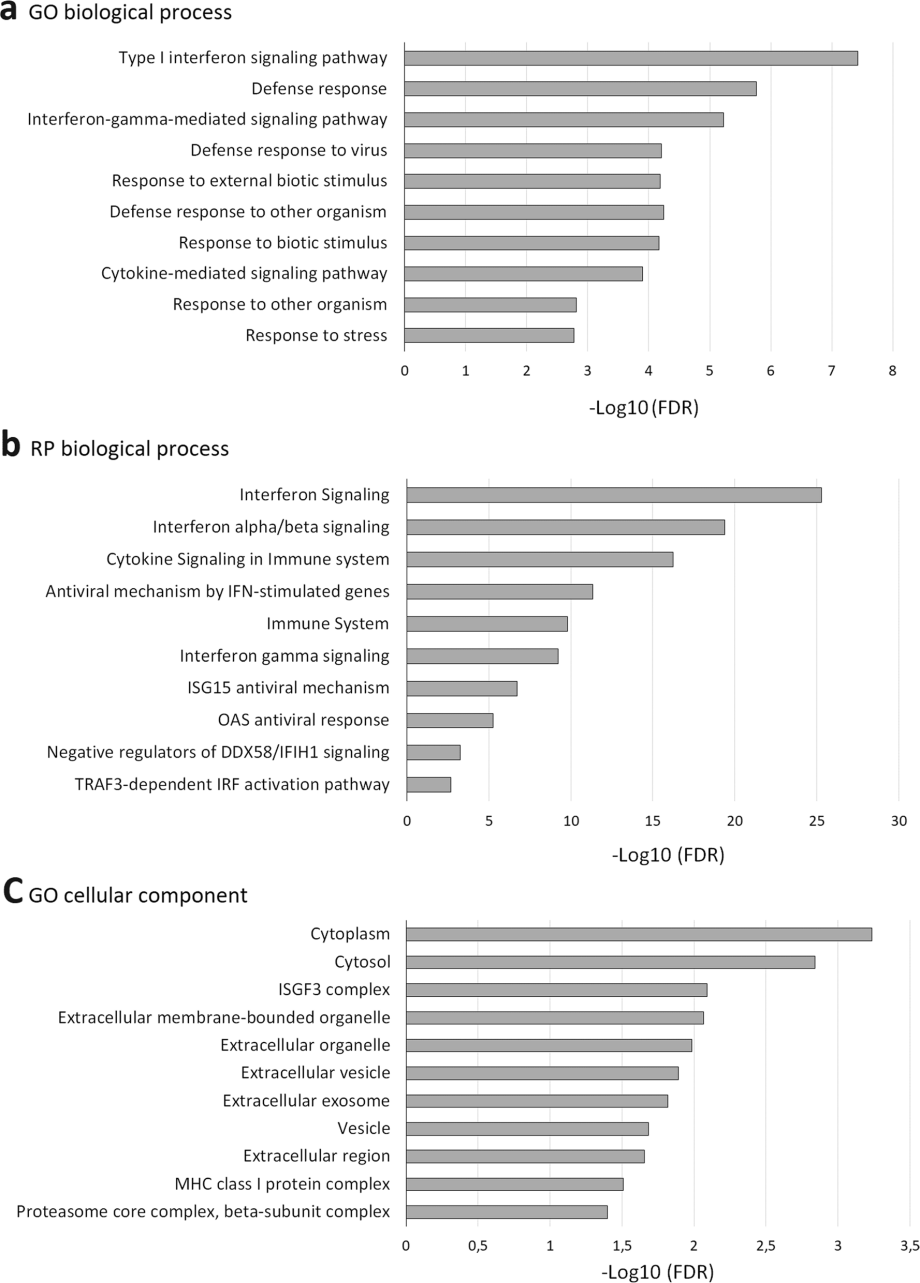
Functional classification of the changed proteins in prMSCs. Biological process in Gene Ontology term (**a**), according to Reactome pathways (**b**) and cellular component in Gene Ontology term (**c**). The top 10 most significant biological processes and localization are presented. Enrichment *p* values were adjusted by Benjamini–Hochberg false discovery rate correction

Simultaneously, differentially expressed proteins in prMSCs were mostly overexpressed or appeared de novo. These proteins were functionally annotated. For this, the following categories were used: biological processes, molecular processes, and the category of cellular localization in the GO and Reactome Pathway databases. Based on the results of the analysis, the 10 most significant groups (*p* < 0.05) were visualized for each category (Fig. 5). By analyzing biological processes in terms of GO, proteins that increased after priming were most significantly associated with type I interferon signaling and a protective response to the virus. Reactome pathway analysis also showed significant regulation (*p* < 0.05) of several signaling pathways associated with interferon signaling and antiviral response. In addition, according to the Reactome, the proteins of the investigated group were also involved in the downregulation of the interferon pathway through DDX58/IFIH1 signaling (pathway identifier: R-HSA-936440).

Overexpressed proteins and proteins identified only in prMSCs were also annotated for molecular function and localization using GO terms. No significant groups related to molecular functions were identified. At the same time, localization annotated proteins were components of the cytosol, the ISGF3 complex, extracellular vesicles, etc. (Fig. 5c).

**Fig. 6:**
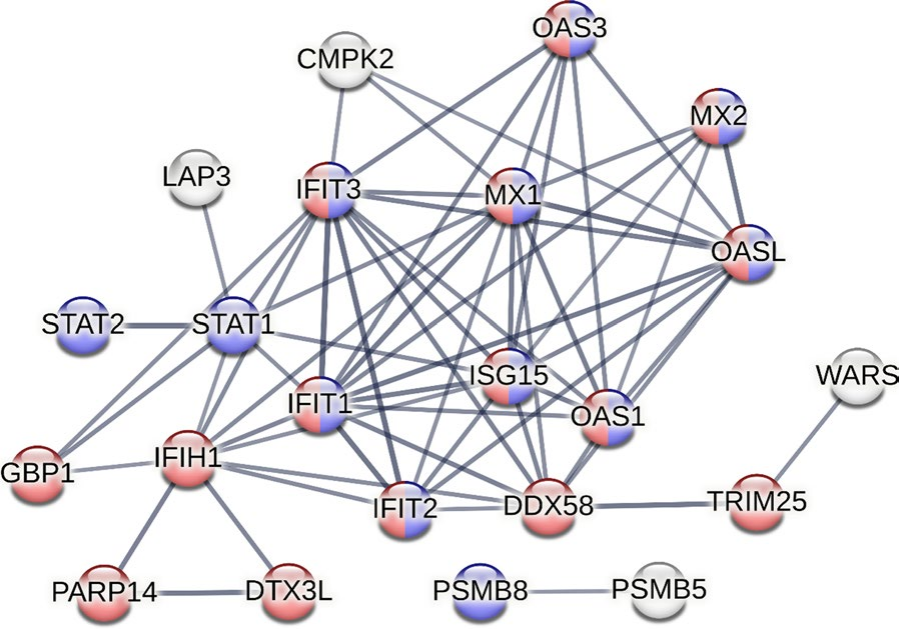
Analysis of STRING interactions for 22 proteins that were more abundant in prMSCs compared to MSCs: the network was built based on high density (0.7), and the edges of the network represent the significance of the interaction. The disabled nodes in the network have been hidden. All networks were enriched using the intersection of 8612 genes present on all platforms as a background, as well as data from experimental protein‒protein interactions, text mining, and curated databases. Proteins from the network of interactions that form the type I interferon signaling cluster according to the biological process (GO) are marked in blue. Proteins associated with interferon alpha/beta signaling and negative regulators of DDX58/IFIH1 signaling are highlighted in red, according to the local network cluster (STRING)

Using GO terms to filter the dataset, we confirmed that proteins induced by poly(I:C) priming were primarily associated with the activation of the immune response. In particular, proteins with the most pronounced activation included several antiviral proteins (STAT1, STAT2, DDX58, GBP1, etc.), as well as those associated with type I IFN signaling (IFIT1, IFIT3, HLA-A, HLA-B, PSMB8, etc.). Protein‒protein interaction analysis of all altered proteins in prMSCs showed that there was a relevant protein interaction between them (Fig. 6).

Analysis of proteins with altered expression in prMSCs showed that the interaction cluster in STRING was formed by de novo proteins (USP18, OAS1/2/3, MX1/2, IFIH1, and IFIT2) and upregulated proteins (other proteins in Fig. 6). This is consistent with the results of functional annotation in terms of GO. The components of the complex were annotated as belonging to processes associated with type I IFN signaling and downregulation of the DDX58/IFIH1 pathway. Overall, our data revealed dynamic changes in the proteome induced by poly(I:C) in MSCs, especially in proteins responsible for enhancing the antiviral response and immunomodulatory functions.

## Discussion

Toll-like receptors are the best-studied pattern recognition receptors that specifically recognize pathogen-associated molecular patterns, such as double-stranded RNA (TLR3). Activation of TLR3 by the poly(I:C) ligand in human macrophages, dendritic cells, and epithelial cells is known to trigger MyD88-independent signaling cascades [35, 36], unlike other TLRs. Activation of TLR3 results in the nuclear translocation of NF-κB and secretion of proinflammatory cytokines, which ultimately leads to the maturation of dendritic cells [37]. Thus, TLR3 modulates the innate and adaptive immune responses.

TLR activation in MSCs can modulate their immune properties. Binding of the TLR3 ligand through the adapter protein TICAM-1/TRIF induces the expression of IFN type I, including in MSCs [19, 36, 38]. At the same time, IFN-α/β expression can be induced by activation of additional cytosolic receptors in the presence of dsRNA: retinoic acid-induced gene-I (RIG-I/DDX58) and melanoma differentiation-associated antigen 5 (MDA5/IFIH1) [37, 39]. It is known that secreted MSCs IFN-β can have an anti-inflammatory effect by increasing the number of T-reg cells [40] and reducing the proliferation of activated T cells when cocultivated with MSCs [19].

Activation of TLRs on effector immune cells (i.e., T and B cells) usually results in the secretion of proinflammatory cytokines [41]. However, MSCs are immunoplastic and, similar to macrophages, can exhibit immunosuppressive or proinflammatory properties, depending on the level of inflammation. This is probably why the priming of TLRs on MSCs can enhance both proinflammatory potency and immunosuppressive properties, depending on the chosen modes of experimental exposure to TLR ligands [17, 19, 42].

T-cell activation is associated with increased tryptophan metabolism. WARS1 and IDO1 are the main enzymes involved in tryptophan metabolism and can be activated in MSCs by the inflammatory environment [28, 43, 44]. Increased production of WARS1 is known to interact with IDO1 to regulate immune responses in vivo; namely, IDO1 catalyzes the degradation of tryptophan into kynurenine, and WARS1 is associated with its transport into cells [45]. It has been shown that TLR3 activation in MSCs leads to STAT1 phosphorylation and subsequent activation of IDO1. The change in the balance between the activity of WARS1 and IDO1 in primed MSCs determines the decrease in the availability of tryptophan for protein synthesis and thus mediates the immunosuppressive effects of MSCs on T cells during their cocultivation [46, 47]. Therefore, in this study, the expression levels of *IDO1* and *WARS1* were assessed during the first optimization stage of the MSC priming protocol.

According to our results, TLR3 priming in all studied modes led to activation of WARS1 (Figs. 2b, 6) and IDO1 (Figs. 2a, 6). We noted that the greatest response to *IDO1* and *WARS1* was observed after 3 h of incubation of cells with 1 and 10 µg/mL poly(I:C). It was first shown that *WARS1* can be activated by poly(I:C), similar to preconditioning with IFN-γ, in MSCs [28]. In addition, our results are consistent with a number of papers in which poly(I:C) exposure resulted in increased *IDO1* expression [17, 19, 48, 49]. However, priming protocols differed in all cases. Thus, Lombardo and DelaRosa noted an increase in IDO1 activity upon stimulation with 10 μg/mL poly(I:C) but not at 1 μg/mL [16]. At the same time, Michalis Mastri et al. showed that exposure to poly(I:C) can trigger differential trophic responses [50]. The contradictions between our results and those of Liotta et al., who did not observe an effect of TLR3 priming on IDO1 activation [51], may be due to differences in the concentration and duration of exposure to the stimulatory ligand. Since TLRs perceive exogenous and endogenous stress signals, the intensity and duration of these signals can differently affect the properties of MSCs [17], which are the so-called "inflammatory switches". Thus, when priming TLR, it is important to normalize the concentration and time of exposure to the ligand, since these parameters can determine the immune status of MSCs.

We also investigated the expression of *TSG-6*, which is involved in extracellular matrix remodeling and is a potent inhibitor of neutrophils [29]. TSG-6 is known to have anti-inflammatory activity through modulation of chemokines/cytokines produced by damaged tissue [52]. As a result, secreted MSCs TSG-6 can suppress the development of Th1 by directly inhibiting the activation of T cells or by suppressing the activation of antigen-presenting cells, disrupting the translocation of NF-κB to the nucleus [53]. To our knowledge, this is the first study to show that TLR3 priming in MSCs leads to an increase in *TSG-6* expression and that the expression level is modulated by the poly(I:C) preconditioning protocol (Fig. 2c). *PD-L1* expression also depended on the activation protocol used (Fig. 2d). PD-1 is expressed on various activated immune cells (including T cells, B cells, macrophages, DCs, etc.), interacting with ligands (PD-L1 and PD-L2), and these activated immune cells are depleted. Thus, the intensity of inflammation decreases in chronic infection and autoimmunity [54]. Increased PD-L1 expression can enhance the immunosuppressive function of MSCs by inducing T regulatory cells and modulating cytokine expression.

Prostaglandin E2 is another key MSC cytokine involved in suppressing the proliferation of activated T cells [18, 55, 56]. MSC treatment with dsRNA activates *PTGES2* and *PGES*, which are involved in PGE2 production [57]. At the same time, the effect of this cytokine on T cells is associated with the inhibition of intracellular calcium release [58], a decrease in the activity of p59 protein tyrosine kinase, and the level of IL-2 secretion [59]. The source of stem cells determines the level of constitutive PGE2 secretion. Intact MSCs from Wharton's jelly express less PGE2 than MSCs isolated from the bone marrow (MSCs-BM) [60] and, as a result, do not suppress T-cell proliferation [61]. This may explain our conflicting results with those of Liotta et al., who did not observe the effect of TLR3 priming on PGE2 levels owing to their high constitutive expression in MSCs-BM [51]. In contrast, in our study, stimulation with 10 μg/ml poly(I:C) for 1 or 3 h led to an increase in *PTGES2* expression and PGE-2 levels, which was consistent with previously obtained data for adipose tissue-derived MSCs (Fig. 2e, f) [55, 56].

The effect of MSCs on cells of the immune system is achieved through direct intercellular interactions, as well as through paracrine signaling. In addition, MSCs isolated from adipose tissue studied in this work have the most significant secretory function compared to MSCs from the bone marrow or umbilical cord [62], and their availability and fewer ethical problems make them more attractive for cell therapy [63]. In this study, a protocol of short-term (3-h) treatment with poly(I:C) (10 µg/mL) resulted in maximum production of anti-inflammatory cytokines and enhancement of the immunosuppressive properties of MSCs, which is consistent with previously published data [17, 18, 49, 59]. However, conflicting results have been reported in a number of works. Thus, Raphaëlle Romieu-Mourez et al*.* reported the formation of a proinflammatory MSC phenotype upon TLR3 priming [20], Liotta et al*.* noted a decrease in immunosuppressive effects [51], and DelaRosa and Lombardo stated that there is no significant effect of poly(I:C) exposure on MSCs modulating immune responses in vitro [25]. As noted earlier, the intensity and duration of TLR3 activating signals can affect MSC properties in different ways. Thus, Raphaëlle Romieu-Mourez et al*.* studied the effect of the culture medium of primed MSCs on peripheral blood mononuclear cells (PBMCs) and primary macrophages. Liotta et al*.* used purified CD4^+^ T cells, while DelaRosa and Lombardo cocultured MSCs with PBMCs and purified CD4^+^ and CD8^+^ T-cell fractions. Simultaneously, in the first case, stimulation was carried out for 6 h at a poly(I:C) concentration of 20 µg/mL, which can lead to increased inflammation [50]. Liotta et al*.* exposed MSCs for 5 days with a TLR agonist (concentration not specified), and DelaRosa and Lombardo evaluated the effect of priming after 72 h of exposure. We noted that long-term exposure to an agonist (24 h) reduced the expression levels of characteristic immunosuppressive markers of MSCs (Fig. 2), which may be associated with the effect of reverse regulation and the decrease/absence of immunosuppressive properties of MSCs. We used linear Jurkat cells to modulate T cell responses, which prevented non-T cells from influencing PBMCs. In this study, we evaluated the effectiveness of various TLR3 stimulation protocols, assuming that MSC immunosuppression is primarily mediated by secreted factors. The optimal exposure was within 3 h of treatment with 10 µg/ml poly(I:C). The enhanced immunosuppressive activity of prMSCs activated according to this protocol was confirmed when MSCs were cocultured with Jurkat cells. It was shown that prMSCs, more actively than MSCs, reduced the proportion of proliferating (by 18%) and increased apoptosis (by 29%) activated T cells but not intact T cells (Fig. 3). T-cell activation leads to increased IL-2 production. PrMSCs inhibited T-cell activation by reducing the expression and secretion of this cytokine (Fig. 3d, f). These data confirm the functionality of MSCs as “inflammatory switches”: TLR3 priming enhances the immunosuppressive effects of MSCs under conditions of inflammation.

Type I IFN signaling is associated with the induction of the immunosuppressive cytokine IL-10 by macrophages [64]. It has been noted that stimulation of TLR3 in MSCs can also increase the secretion of this cytokine [18]. However, the mechanisms underlying the direct immunosuppressive effects of IL-10 are not fully understood [65, 66]. IL-10 is known to inhibit the activation of CD4^+^ T cells by suppressing the production of IL-2 and signaling CD28 [67], and myeloid cells treated with IL-10 lose their ability to respond to lipopolysaccharide (LPS) [68]. We showed that when cocultivated with J^+^ prMSCs and MSCs, the level of IL-10 in the medium did not differ but was higher than that after cocultivation with J^−^ and in intact T cells, similar to the data of Yang et al. [69] (Fig. 3e). It is likely that the secretion of this cytokine can be blocked by newly synthesized IL-10 [33], thus limiting its amount in a conditioned environment [70]. It is also known that IL-10 acts at low doses and mainly locally to induce immune responses. We also did not detect the secretion of IL-10 by intact MSCs or prMSCs, similar to the results of Lombardo et al. [16]. At the same time, it was previously shown that IL-10, as a component of the MSC secretome, plays the role of an immunosuppressant, reducing the secretion of IL-2 by activated Jurkat cells [33], and may also be associated with their differentiation into T-reg cells [34]. In addition, increased secretion of IL-10 by transduction of MSCs with the MIG retroviral vector (MSCV-IRES-GFP) or transfection of IL-10 mRNA increases their anti-inflammatory effect in a model of acute graft-versus-host disease or acute respiratory distress syndrome [70, 71].

In the next stage, we performed a proteomic study of MSCs after TLR3 priming according to the chosen protocol and compared the results with those for intact cells. Poly(I:C) mimics dsRNA released during viral replication; thus, TLR3 stimulation activates interferon regulatory factor 3 (IRF3). Activation of IRF3 leads to an increase in the expression of primary response genes (interferon-β (IFN-β), interferon-stimulated gene 15 (ISG15), etc.), which in turn initiates autocrine/paracrine activation of secondary genes (MX1, MX2) [72]. This pathway plays a key role in the antiviral response of cells. In this study, the impact of poly(I:C), according to the results of a proteomic study, led to the activation of ISG15, ISG56 (IFIT1), ISG54 (IFIT2), ISG60 (IFIT3), MX1 and MX2, which are necessary to maintain the viability of MSCs. However, IFIT1 can also negatively regulate the expression of TNF-α and other inflammatory cytokine genes in LPS-treated macrophages while simultaneously stimulating IFN-β expression and the subsequent interferon gene program. It is likely that IFIT1 regulates an important balance between the inflammatory and IFN gene programs to promote an optimal innate immune transcriptional response to microbial infection [73]. The chronic type I IFN signature is also known to increase the expression of PD-L1 and IL-10 in dendritic cells and macrophages, modulating the anti-inflammatory environment [74]. Thus, the level and duration of viral load may influence the immunomodulatory properties of MSCs. We noted a decrease in the number of proliferating T cells after coculture with prMSCs, probably due to the functioning of IFIT1. The TLR3 activation protocol obtained in this study allowed maintenance of the immunosuppressive status of cells.

In addition, exposure to poly(I:C) can lead to the activation of IRF3 through the cytosolic receptors RIG-I/DDX58 and MDA-5/IFIH1 in MSCs [75–77]. Activation of RIG-I strongly induces the OASL protein, which stimulates the production of type I IFN and its antiviral response [78, 79]. STAU1 expression, which we obtained from the results of proteomic analysis, is known to positively regulate the expression of immune response genes (IFIT2, IFIT3, and OASL) [80], and its knockdown leads to inhibition of the activation of the porcine IFN-β promoter [81]. TRIM25, in turn, mediates both RIG-I/DDX58 and MDA-5/IFIH1 ubiquitination and is required for RIG-I-mediated interferon production and antiviral activity [82]. It is important to note that activation of RIG-I/DDX58 and MDA-5/IFIH1 modulates the expression of proinflammatory cytokines, such as IL-6 [77], and immunosuppressive molecules, such as IDO1 [83]. We noted increased expression of *IDO1* earlier in the qRT‒PCR results, and IDO1 was also present in the proteome results as a protein that appeared in prMSCs after exposure to poly(I:C). Thus, we showed that TLR3 functions normally in MSCs, as its activation under the conditions of the obtained protocol triggers type I IFN signaling cascades.

However, type I IFN, along with stimulating antiviral functions, limits damaging immune responses that can lead to tissue pathology and excessive tissue damage. MSCs play a key role in tissue repair and must remain viable to perform their functions. We noted an increase in the amount of DNJA1 protein, which negatively regulates BAX translocation from the cytosol to the mitochondria in response to cellular stress and thus probably additionally protects cells from apoptosis [84]. The inflammatory environment appears to lead to oxidative stress (OS) in MSCs, as we observed an increase in GFTP2 protein, a marker of OS [85]. At the same time, activation of TLR signaling also greatly increases the expression of the antioxidant defense and DNA repair protein SOD2 [86]. We also observed an increase in PNPT1 protein, the import function of which is known to be necessary for mitochondrial respiration and cell viability [87], and TMX, which plays an important role in host defense against OS-related inflammatory conditions [88]. In addition, an increase in LONP1 protease promotes the degradation of oxidized proteins in the mitochondrial matrix, thereby providing increased cell protection [89]. Simultaneously, increased GBP1 expression promotes the migration and invasion of stem cells (human periodontal ligament), which is important for the realization of their regenerative properties. Knockdown of GBP1 in MSCs-BM significantly inhibits the expression of *IDO1*, an important mediator of their immunosuppressive effects [90].

Different states of inflammation can lead to markedly different MSC responses, which indicates the plasticity of their immunomodulation. It has been reported that graft-versus-host disease can be successfully treated with MSCs administered in severe inflammation, but efficacy is reduced if MSCs are administered on the day of bone marrow transfusion, that is, before the onset of inflammation [91, 92]. Similarly, the therapeutic effects of MSCs in experimental autoimmune encephalomyelitis were reduced when the cells were administered during disease remission [93, 94]. Thus, the inflammatory state appears to influence the immunosuppressive effect of MSCs. Simultaneously, the inflammatory status changes throughout the immune response. In the presence of the TLR3 ligand, we observed an increase in proteins associated with antiviral protection, maintenance of MSC viability, and mediation of anti-inflammatory responses.

MSCs activated by inflammatory cytokines (IL-1, TNF-α, and IFN-γ) express adhesion molecules, such as intercellular adhesion molecule 1 (ICAM-1) and vascular cell adhesion molecule (VCAM-1), to enhance leukocyte binding [95]. Chemokines and adhesion molecules attract and attach T cells to MSCs, and high concentrations of immunosuppressive effector molecules can act on T cells and suppress their proliferation [96]. We have shown for the first time that TLR3 priming under conditions of short-term exposure (3 h) at a concentration of 10 µg/mL poly(I:C) leads to the appearance of ICAM-1 protein on the MSC surface. We also noted enhanced adhesion of Jurkat cells to prMSCs during cocultivation relative to intact MSCs (data not shown). In addition, we noted the appearance of galectin-3 protein (LG3BP) upon treatment with poly(I:C) MSCs, which promotes integrin-mediated cell adhesion. It has previously been shown that LG3BP production was also significantly increased upon stimulation of TLR2 by a ligand [97]. Binding of galectin-3 to receptor kinases, such as CD45 and T-cell receptors, is critical for the regulation of their function. Galectin-3 is also involved in suppressing immune surveillance in tumors by killing T cells and interfering with NK cell function [98]. The immunosuppressive effect of MSCs has previously been shown to be mediated through the secretion of galectin-3, which modulates T-cell proliferation, cell adhesion and migration [99]. These data confirm the enhanced immunosuppressive effects of MSCs obtained in this study due to cellular contact with immune cells.

In addition, priming has been shown to increase poly(ADP-ribose) polymerase family member 14 (PARP14) protein, which is known to suppress IFN-γ-induced macrophage activation [100] and plays an immunosuppressive role in tumors [101]. PARP14 is an important cofactor for signal transduction and transcriptional activation 6 (STAT-6), which is involved in the differentiation of IL-10-secreting Th2 cells [102]. PARP14 also promotes cell survival by inhibiting caspase activity [103]. We observed an increase in proteasome 20S subunit beta 8 (PSMB8) protein levels in the prMSCs. Inhibition of the PSMB8 immunoproteasome can induce inflammatory reactions [104]. PSMB8 overexpression reduces the virus-induced activation of NF-κB promoters and suppresses the expression of proinflammatory IL-1β, TNF-α, IL6, IL8, and IFN-γ upon infection with nerve necrosis virus [105]. It is also known that USP18 is a negative regulator of TLR-mediated NF-κB activation, particularly in human macrophages. USP18 overexpression also inhibits the secretion of IL-1β, IL-6, TNF-α, and IL-18 in LPS-induced human pulmonary microvascular endothelial cells. USP18 knockdown enhances the expression of proinflammatory cytokines in macrophages [106]. Thus, USP18 may bind to the NF-κB and type I IFN signaling pathways to avoid an excessive inflammatory immune response [106]. An increase in GBP4 in prMSCs likely regulates the excess production of type I IFN, since it targets IRF7 and inhibits its transactivation [107]. In addition, we noted Nectin-2 protein in the proteome results, which is a modulator of T-cell signaling. Nectin-2 can be a costimulator of T-cell function or a coinhibitor, depending on its receptor. On the one hand, it stimulates T-cell proliferation and the production of cytokines, including IL2, IL5, IL10, IL13, and IFN-γ, after CD226 binding. On the other hand, it inhibits T-cell proliferation after interacting with poliovirus receptor-related immunoglobulin domain-containing (PVRIG). These interactions are competitive [108]. Thus, we suggest that the immunosuppressive effects of MSCs are achieved, among other things, due to the appearance of PARP14, PSMB8, and Nectin-2 proteins, as well as the immunomodulatory roles of USP18 and GBP4.

## Conclusions

TLR3 activation conditions in MSCs influence their immunosuppressive abilities. Preconditioning MSCs with 10 μg/mL poly(I:C) for 3 h induced the maximal expression of *IDO1*, *WARS1*, *PD-L1*, *TSG-6*, *PTGES2*, and PGE2 secretion. Proteomic analysis revealed changes in proteins related to the immunosuppressive properties of prMSCs, such as ICAM-I, Galectin-3, PARP14, PSMB8, USP18, and GBP4. The TLR3 priming protocol did not alter the immunophenotype of MSCs. PrMSCs had stronger immunosuppressive effects on activated T cells than naive MSCs, reducing Jurkat cell activation and proliferation and increasing apoptosis. The proposed MSCs preconditioning strategy enhanced the anti-inflammatory properties and could be used to overcome the heterogeneity of the cell population. Therefore, the response to cell therapy with prMSCs for autoimmune or inflammatory diseases may be more effective and predictable. However, the mechanisms underlying the immunosuppressive effects of the proteins identified by proteome profiling in prMSCs should be thoroughly investigated prior to preclinical testing through functional inactivation or overexpression studies.

### Supplementary Information

**Additional file 1: Table S1**. Primers used in the study

## Data Availability

The mass spectrometry proteomics data have been deposited to the ProteomeXchange Consortium via the PRIDE partner repository [109] with the dataset identifier PXD044795.

## Abbreviations

ELISA: Enzyme-linked immunosorbent assay
FDR: False positive identifications
GBP4: Guanylate-binding protein 4
GO: Gene Ontology
ICAM-I: Intercellular adhesion molecule 1
IDO1: Indoleamine 2,3-dioxygenase
IFN: Interferon
IL: Interleukin
IRF3: Interferon regulatory factor 3
J^−^: Nonactivated Jurkat cells
J^+^: Activated Jurkat cells
LC‒MS: Liquid chromatography–mass spectrometry
LFQ: Label-free quantification
LG3BP: Galectin-3
LPS: Lipopolysaccharide
MDA5/IFIH1: Melanoma differentiation-associated antigen 5
MSCs: Mesenchymal stromal cells
NK: Natural killer
OS: Oxidative stress
PARP14: Poly(ADP-ribose) polymerase family member 14
PBMCs: Peripheral blood mononuclear cells
PD-L1: Programmed cell death 1 ligand 1
PGE-2: Prostaglandin E2
PHA: Phytohemagglutinin
PI: Propidium iodide
PMA: Phorbol myristyl acetate
Poly(I:C): Polyinosinic-polycytidylic acid
PrMSCs: MSCs with activated TLR3
PSMB8: Proteasome 20S subunit beta 8
PTGES2: Prostaglandin-endoperoxide synthase 2
PVRIG: Poliovirus receptor-related immunoglobulin domain-containing
qRT‒PCR: Quantitative real-time PCR analysis
RIG-I/DDX58: Retinoic acid-induced gene-I
SEM: Standard error of the mean
TEAB: Triethylammonium bicarbonate buffer
Th: T-helper cell
TLRs: Toll-like receptors
TNF-α: Tumor necrosis factor-alpha
TSG-6: TNF-α–stimulated gene 6
USP18: Ubl carboxyl-terminal hydrolase 18
VCAM-1: Vascular cell adhesion molecule 1
WARS1: Tryptophanyl-tRNA synthetase 1
