# Supplementary material for: The effect of TLR3 priming conditions on MSC immunosuppressive properties
Source: Stem Cell Res Ther. 2023 Nov 29;14:344. doi: 10.1186/s13287-023-03579-y (PMC10687850; doi:10.1186/s13287-023-03579-y)
Supplement: Supplementary file 1 — Additional file 1: Table S1. Primers used in the study [file 13287_2023_3579_MOESM1_ESM.docx]

***Table S1. Primers used in the study***

| Gene | Forward | Reverse |
| --- | --- | --- |
| *IDO1* | CCCTTCAAGTGTTTCACCAAATC | GTCTTCCCAGAACCCTTCATAC |
| *WARS1* | CAGTACAGACAAGCAGTGCAAA | GGTGGCTCTCTCTATTCGGTTTA |
| *TSG-6* | AAGATGGGATGCCTATTGCTAC | ATTTGGGAAGCCTGGAGATTTA |
| *PD-L1* | GCACACTGAGAATCAACACAAC | AGTCCTTTCATTTGGAGGATGT |
| *PTGES2* | CAGCACTTCACGCATCAGTT | GTCTAGCCAGAGTTTCACCGTA |
| *IL-2* | CAGCAATATCAACGTAATAGTTCTGGA | AGTCAGTGTTGAGATGATGCTTTG |
| *ACTB* | TCAGAAGGATTCCTATGTGGGCGA | CACGCAGCTCATTGTAGAAGGTGT |
| *GAPDH* | TCGACAGTCAGCCGCATCTTCTTT | ACCAAATCCGTTGACTCCGACCTT |
